# A Retrospective Study of the Relationship Between Blood Transfusion and 30-Day Postoperative Outcomes in Patients Undergoing Isolated Off-Pump Coronary Artery Bypass Grafting

**DOI:** 10.21470/1678-9741-2021-0031

**Published:** 2022

**Authors:** Liangyuan Lu, Ji Che, Weiping Cheng, Ran Dong, Jiapeng Huang, Zhanmin Yang, Jiakai Lu

**Affiliations:** 1 Department of Anesthesiology, Aerospace Center Hospital, Beijing, People’s Republic of China; 2 Department of Blood Transfusion, Beijing An Zhen Hospital, Capital Medical University, Beijing Institute of Heart, Lung and Blood Vessel Diseases, Beijing, People’s Republic of China; 3 Department of Anesthesiology, Beijing An Zhen Hospital, Capital Medical University, Beijing Institute of Heart, Lung and Blood Vessel Diseases, Beijing, People’s Republic of China; 4 Department of Cardiac Surgery, Beijing An Zhen Hospital, Capital Medical University, Beijing Institute of Heart, Lung and Blood Vessel Diseases, Beijing, People’s Republic of China; 5 Department of Anesthesiology & Perioperative Medicine, University of Louisville, Louisville, Kentucky, United States of America

**Keywords:** Myocardial Infarctation, Coronary Artery Bypass, Off-Pump, Erythrocytes, Propensity Scores, Intensive Care Unit, Survival Rate, Mortality

## Abstract

**Introduction:**

The objective of this single-center study it to retrospectively analyze the relationship between transfusion and 30-day postoperative outcomes in patients undergoing isolated off-pump coronary artery bypass grafting.

**Methods:**

Perioperative data of 2,178 patients who underwent isolated off-pump coronary artery bypass grafting from 2018 to 2019 were collected. A 1:1 propensity score matching was performed to control for potential biases between patients who received blood transfusion and those who did not. After propensity score matching, we analyzed the clinical outcomes of transfusion and non-transfusion patients. Postoperative complications and the survival of patients within 30 days after surgery in both groups were analyzed. Kaplan-Meier survival curve and log-rank test were used for survival analysis.

**Results:**

The total blood transfusion rate of all patients was 29%, including red blood cell (27.6%), plasma (7.3%), and platelet (1.9%). Four hundred and forty patients in each group were compared after propensity score matching. There were no significant differences in the incidence of stroke, myocardial infarction, atrial fibrillation, acute kidney function injury, and sternal wound infection of both groups (P>0.05). However, higher incidence of postoperative pulmonary infection and more mechanical ventilation time and days of stay in the intensive care unit and postoperative in-hospital stay were associated with blood transfusion (P<0.05). The 30-day cumulative survival rate of the transfusion group was lower than that of the control group (P<0.05).

**Conclusion:**

Perioperative blood transfusion increases the risks of postoperative pulmonary infection and short-term mortality in off-pump coronary artery bypass grafting patients.

**Table:** 

Abbreviations, Acronyms & Symbols				
AF	= Atrial fibrillation		LVEDD	= Left ventricular end-diastolic diameter
AKI	= Acute kidney injury		LVEF	= Left ventricular ejection fraction
BMI	= Body mass index		MI	= Myocardial infarction
CABG	= Coronary artery bypass grafting		NSAIDs	= Non-steroidal anti-inflammatory drugs
CI	= Confidence interval		NYHA	= New York Heart Association
COPD	= Chronic obstructive pulmonary disease		OPCABG	= Off-pump coronary artery bypass grafting
CPB	= Cardiopulmonary bypass		OR	= Odds ratio
FIB	= Fibrinogen		PSM	= Propensity score matching
HF	= Heart failure		RBC	= Red blood cell
IABP	= Intra-aortic balloon pump		SCr	= Serum creatinine
ICU	= Intensive care unit		Transf.	= Transfusion
INR	= International normalized ratio			

## INTRODUCTION

Blood transfusion can save lives, but like all therapeutics, it also carries risks and costs. According to the Society of Thoracic Surgeons and the Society of Cardiovascular Anesthesiologists blood conservation clinical practice guidelines, 50% of heart surgical patients receive blood transfusion treatment^[1]^. The rational use of blood products is usually a concern for medical institutions around the world.

Coronary artery bypass grafting (CABG) performed with the assistance of cardiopulmonary bypass (CPB) procedure is still the dominant treatment for complicated coronary artery diseases^[1]^. Allogeneic blood transfusion was generally considered to be closely related to the outcomes of surgical patients. Patients who received blood transfusion for cardiac surgery were considered to be strongly associated with postoperative complications and mortality^[2,3]^.

The relationship between blood transfusion and postoperative complications in cardiac surgery was reported in many single or multi-center studies. But the results are still inconsistent^[3,4]^. As our hospital is currently the second largest center of cardiac surgery in China, with annual volume over 15,000 cases of cardiac surgery, and the amount of blood used is enormous, we conducted a retrospective study in patients undergoing isolated off-pump CABG (OPCABG) during one year by reducing errors due to differences caused by time and institutions. The primary aim of this study was to analyze the relationship between blood transfusion and 30-day postoperative clinical outcomes.

## METHODS

This study has retrospectively analyzed the patients who underwent isolated OPCABG from April 1, 2018 to March 31, 2019. We collected information of perioperative blood transfusion including red blood cell (RBC), plasma, and platelet and tried to examine the association between clinical outcomes and blood transfusion in these patients. Because this set of data was extracted from the institution’s database, we conducted an observational study by applying propensity score matching (PSM) to form groups for comparison with near identical distributions of background and potential confounder variables. After PSM, we analyzed the clinical outcomes of transfusion and non-transfusion patients undergoing OPCABG surgery.

### Ethics Committee

Ethics approval was obtained from the An Zhen Hospital’s ethics committee (2022004X). The Ethics Review Board waived the requirement of obtaining patients’ informed consent because the study was retrospective in nature and identification of patients was not necessary.

### Data Source

The authors collected the records of patients who underwent isolated OPCABG in the An Zhen Hospital in one year, from April 2018 to March 2019. Patients were excluded for any of the following reasons: age < 18 years, pregnancy, combined surgery, and patients who underwent CPB or converted from OPCABG to on-pump CABG during surgery.

Standard demographic and clinical characteristics were obtained from the institutional clinical database. Detailed perioperative data included demographics, comorbidities, New York Heart Association (NYHA) class, emergency status, laboratory tests, imaging and ultrasound examination reports, blood transfusion compositions (RBC, plasma, platelet), type of surgery, operation time, treatments during operation, intra-aortic balloon pump (IABP), intraoperative and postoperative blood loss, re-exploration for hemostasis, postoperative complications, and survival within 30 days after surgery. All queries were resolved by referring to the patients’ original records. Missing data fields that could not be obtained from the original records were replaced by the mean of each subgroup (< 0.5% in total study population). In addition to a random audit of 10% of all cases, patients who died were reviewed routinely to ensure data accuracy. The audit has revealed a data accuracy > 99% for the study population.

### Definitions

Patients who received any allogeneic blood transfusion during perioperative period were defined as transfusion group, and those who did not receive allogeneic transfusion were defined as control group.

Acute kidney injury (AKI) was defined as increased serum creatinine (SCr) by ≥ 0.3 mg/dl (≥ 26.5 µmol/L) within 48 hours or increased SCr to ≥ 1.5 times baseline, which was known or presumed to have occurred within the first seven days after surgery.

Stroke was clinically identified as a new persistent neurological deficit, such as any adverse event including cerebrovascular accident, cerebral embolism, cerebral hemorrhage, cerebral infarct, or cerebral ischemia.

Myocardial infarction (MI) was defined as either a new Q-wave, development of a new and persistent left bundle branch block, or creatine kinase-myocardial band > 5 times upper limit of normal after surgery. Heart failure (HF) was defined as dyspnea at rest and the presence of at least one of the following symptoms: pulmonary edema, signs of pulmonary congestion on X-ray, need for continuous positive airway pressure or mechanical ventilation, or need for intravenous diuretics due to symptoms of congestion or persistent oliguria (urine output < 0.5 mL/kg/h) after volume therapy.

Pulmonary infection was diagnosed by multidisciplinary consultation according results of pulmonary imaging examination and laboratory tests after surgery. Patients were diagnosed with sternal wound infection occurring within the period from surgery through postoperative 30 days.

The information in the medical records of patients who died in 30 days after surgery for any reasons was collected and double-checked.

Intraoperative and postoperative RBC transfusions were administrated if hemoglobin concentration was < 8 g/dl during surgery or < 9 g/dl in the intensive care unit (ICU). The patient’s blood transfusion was determined by anesthesiologist during surgery or intensive physician in the ICU, according to the blood management policy in our hospital supervised by the department of transfusion. Autologous blood transfusion (intraoperative blood salvage procedure) was applied in all patients in the study.

### Statistical Analysis

A 1:1 PSM was performed to control for potential biases between patients who received blood transfusion and who did not. According to the outcome variables (transfusion) and confounding variables, a binary logistic regression model was established by stepwise regression method. Covariates were screened according to the regression results. Variables that were included in the model were selected into covariates (*P*<0.05), while those that were excluded were selected into additional covariate. The caliper value of PSM was set as 0.1. The standard deviation was used to evaluate the difference in both groups before and after PSM. Standard deviation < 10% was regarded as the baseline feature matching of the two groups.

Summary variables were expressed as frequencies (percentages) and compared between patient groups using the chi-squared or Fisher’s exact tests. Shapiro-Wilk tests were used to determine the distribution of continuous variables. Normally distributed data were reported as mean ± standard deviation and compared between groups using Student’s *t*-test. Skewed data were expressed as median (interquartile range) and compared between groups using Wilcoxon rank sum tests. Kaplan-Meier curve was used to calculate the cumulative survival rate. Log-rank test was used for survival analysis.

Statistical analyses were performed with computer software IBM Corp. Released 2013, IBM SPSS Statistics for Windows, version 22.0, Armonk, NY: IBM Corp. All *P*-values were two sided, and *P*<0.05 was considered statistically significant.

## RESULTS

**Fig. 1:**
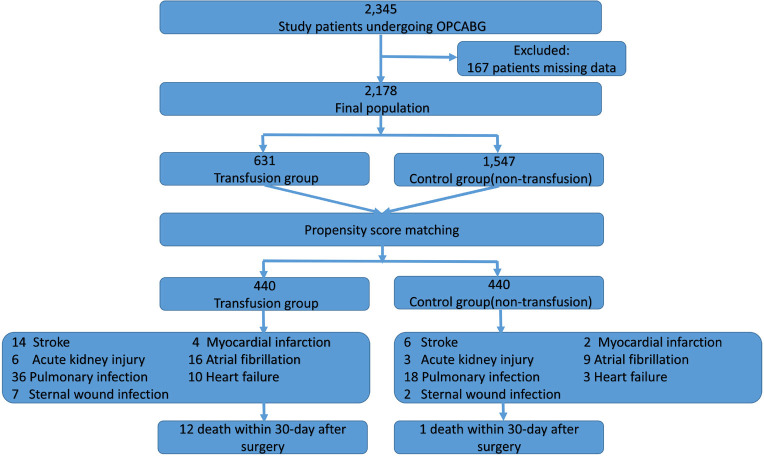
Flowchart of selection process. A total of 2,345 patients were screened and 2,178 patients entered the final analysis; 167 patients were excluded because of missing important data from original database, including imaging examinations, medical history, records during operation, and treatments after surgery. OPCABG=off-pump coronary artery bypass grafting.

**Fig. 2:**
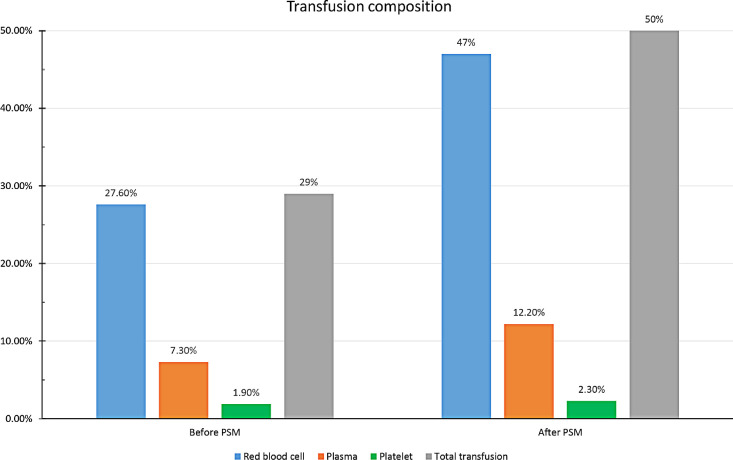
Composition of transfusion in patients undergoing off-pump coronary artery bypass grafting. Red blood cell, plasma, and platelet represent the compositon of transfusion in proprotion of transfusion group. Total transfusion means proportion of the total number of patients receiving blood transfusion. PSM=propensity score matching.

A total of 2,345 patients were screened, and 167 patients were excluded because of missing important data from original database, including imaging examinations, medical history, records during operation, and treatments after surgery. Patients with complete information were divided into two groups, including 631 patients in the transfusion group and 1,547 patients in the control group (Figure 1). A 1:1 PSM was performed to control for potential biases, including sex, age, body mass index (BMI), medical history, perioperative examination, intraoperative variables, postoperative variables, emergency status, and discontinuation of aspirin (> 7 days), between patients who received blood transfusion and those who did not. The missing data replaced with the mean of each subgroup of two groups were also analyzed in the propensity matching procedure. After PSM, 440 patients were finally enrolled in each group. The total perioperative transfusion rate was 29% approximately, including RBC (27.6%), plasma (7.3%), and platelet (1.9%), before PSM (Figure 2). When preoperative and intraoperative confounders were adjusted, composition of blood transfusion was 47% of RBC, 12.2% of plasma, and 2.3% of platelet in the transfusion group.

Characteristics of the patients undergoing OPCABG were compared in both groups before PSM (Table 1). Baseline data of perioperative characteristics, including gender, age, BMI, diabetes, hyperlipidemia, smoking history, NYHA class, hemoglobin, alanine transaminase, blood glucose, fibrinogen, left ventricular end-diastolic diameter, IABP, sequential vein bypass grafting, use of internal mammary artery, operation time, dose of heparin, discontinuation of aspirin (> 7 days), intraoperative and postoperative blood loss, and re-exploration for hemostasis, were significantly different in both groups (*P*<0.05). There were no statistical differences between characteristics of the transfusion group and the control group after PSM (*P*>0.05).

**Table 1:** Comparison of baseline characteristics before and after propensity score matching (PSM) between transfusion (Transf.) group and control group.

Characteristic		Before PSM			After PSM		
		Transf. group (n=631)	Control group (n=1547)	*P*-value	Transf. group (n=440)	Control group (n=440)	*P*-value
Female		257 (40.7)	280 (18.1)	< 0.001	159 (36.1)	166 (37.7)	0.625
Age (years)		65.2±0.3	61.9±0.2	< 0.001	64.2±0.4	64.2±0.4	0.874
BMI (kg/m2)		25.1±0.1	29.4±3.4	< 0.001	25.3±0.1	25.5±0.2	0.627
NYHA (III/IV)		273 (43.3)	509 (32.9)	< 0.001	162 (36.8)	176 (40)	0.332
Medical history	Hypertension	420 (66.6)	1005 (65)	0.477	293 (67)	292 (66.4)	0.943
	Diabetes	277 (43.9)	578 (37.4)	0.005	186 (42.3)	190 (43.2)	0.785
	Hyperlipidemia	334 (52.9)	701 (45.3)	0.001	224 (50.9)	218 (49.5)	0.686
	Smoking	227 (36)	797 (51.5)	< 0.001	180 (40.9)	178 (40.5)	0.891
	Cerebrovascular disease	115 (18.2)	266 (17.2)	0.566	71 (16.1)	69 (15.7)	0.854
	COPD	20 (3.2)	44 (2.8)	0.683	16 (3.6)	20 (4.5)	0.496
	Acute MI	130 (20.6)	267 (17.3)	0.067	84 (19.1)	75 (17)	0.430
	Coronary stents before operation	84 (13.3)	232 (15)	0.311	65 (14.8)	61 (13.9)	0.700
	AF	11 (1.7)	20 (1.3)	0.421	7 (1.6)	8 (1.8)	0.795
	Heart failure	8 (1.3)	9 (0.5)	0.099	4 (0.9)	4 (0.9)	1.000
Perioperative examination	Hemoglobin (g/L)	132.3±0.6	143.3±0.3	< 0.001	136.7±0.7	137.3±0.6	0.766
	Platelet count (109/L)	226.2±2.6	227.1±1.5	0.552	226.1±3.1	225.6±2.7	0.749
	Alanine transaminase (µ/L)	31.7±2.6	33.7±0.7	< 0.001	30.6±1.3	32.2±1.3	0.199
	Creatinine (mmol/l)	83.9±3.4	74.5±0.8	0.078	74.6±1.3	74.9±2.3	0.196
	Blood glucose (mmol/l)	7.2±0.1	6.9±0.1	0.037	7.1±0.1	7.1±0.1	0.441
	Triglyceride (mmol/l)	1.82±0.07	1.71±0.03	0.437	1.82±0.07	1.71±0.05	0.845
	INR	1.02±0.01	1.02±0.01	0.591	1.02±0.01	1.02±0.01	0.542
	Plasma FIB (g/L)	3.42±0.03	3.34±0.02	0.023	3.38±0.04	3.40±0.04	0.842
	LVEF (%)	59.3±0.3	60.1±0.2	0.136	59.9±0.4	59.7±0.4	0.686
	LVEDD (mm)	47.2±0.2	48.5±0.1	< 0.001	47.5±0.3	47.7±0.3	0.282
Intraoperative variables	IABP	144 (22.8)	106 (6.9)	< 0.001	69 (15.7)	73 (16.6)	0.714
	Internal mammary artery	417 (66.1)	1260 (81.4)	< 0.001	328 (74.5)	319 (72.5)	0.492
	Sequential vein bypass grafting	559 (88.6)	1369 (88.5)	< 0.001	390 (88.6)	398 (90.5)	0.513
	Number of grafts	3.65±0.03	3.58±0.02	0.135	3.63±0.04	3.66±0.04	0.440
	Operation time (hours)	4.36±0.04	4.18±0.02	< 0.001	4.31±0.04	4.29±0.04	0.441
	Urgent operation	40 (6.3)	76 (4.9)	0.179	30 (6.8)	26 (5.9)	0.581
	Local hemostatic	487 (77.2)	1216 (78.6)	0.465	346 (78.6)	353 (80.2)	0.559
	Dose of heparin (mg)	117.3±1.5	118.5±0.7	0.002	117.2±1.6	115.0±1.3	0.581
	Tranexamic acid	328 (52)	960 (62.1)	< 0.001	248 (56.4)	242 (55)	0.684
	Intraoperative blood loss (ml)	895.1±421.9	734.2±280.2	< 0.001	887.4±414.7	825.1±555.1	0.060
Postoperative variables	Postoperative blood loss (ml)	633.2±464.2	407.7±240	< 0.001	656.7±472.4	619.3±233.4	0.137
	Re-exploration for hemostasis	26 (4.1)	7 (0.5)	< 0.001	5 (1.1)	2 (0.5)	0.448
	Emergency status	40 (6.3)	76 (4.9)	0.179	30 (6.8)	26 (5.9)	0.581
	Discontinuation of aspirin (> 7 days)	483 (76.5)	1318 (85.2)	< 0.001	355 (80.7)	350 (79.5)	0.673

AF=atrial fibrillation; BMI=body mass index; COPD=chronic obstructive pulmonary disease; FIB=fibrinogen; IABP=intra-aortic balloon pump; INR=international normalized ratio; LVEDD=left ventricular end-diastolic diameter; LVEF=left ventricular ejection fraction; MI=myocardial infarction; NYHA=New York Heart Association

**Fig. 3:**
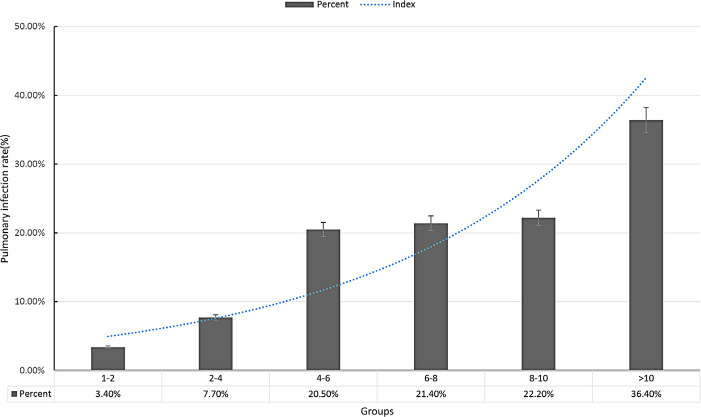
Relationship between red blood cell transfusion and postoperative pulmonary infection. Groups were defined as the amount of red blood cell transfusion. Trend of patients diagnosed with pulmonary infection after surgery increased with the number of allogeneic red blood cell transfusion.

In comparison of the postoperative complications of patients in both groups (Table 2), there were statistical differences in stroke, MI, atrial fibrillation (AF), HF, pulmonary infection, and sternal wound infection before PSM (*P*<0.001). After adjusting for baseline covariates, higher incidence of postoperative pulmonary infection, longer duration of mechanical ventilation, more days of stay in ICU, and higher rate of mortality within 30 days after surgery were associated with transfusion (Tables 3 and 4). The rate of pulmonary infection was 8.2% in the transfusion group compared with 4.1% in the control group. The risk of postoperative pulmonary infection in the transfusion group was two-fold times higher compared with the non-transfusion group. Adjusted odds ratio (OR) estimate was 2.089 for the association of transfusion with pulmonary infection. The relationship between RBC transfusion and pulmonary infection was showed in Figure 3.

**Table 2:** Comparison of clinic outcomes before propensity score matching (PSM) between transfusion (Transf.) group and control group.

Outcomes	Before PSM			
	Transf. group (n=631)	Control group (n=1547)	Odds ratio (95% CI)	*P*-value
Stroke	21 (3.3)	12 (0.8)	4.404 (2.153-9.006)	< 0.001
Myocardial infarction	10 (1.6)	3 (0.2)	8.288 (2.273-30.215)	< 0.001
Atrial fibrillation	35 (5.5)	24 (1.6)	3.727 (2.198-6.319)	< 0.001
Heart failure	28 (4.4)	5 (0.3)	14.320 (5.504-37.259)	< 0.001
AKI	10 (1.6)	11 (0.7)	2.249 (0.950-5.321)	0.058
Pulmonary infection	67 (10.6)	64 (4.1)	2.753 (1.928-3.930)	< 0.001
Sternal wound infection	10 (1.6)	5 (0.3)	4.966 (1.691-14.588)	0.001
Death within 30 days after surgery	20 (3.2)	5 (0.3)	10.095 (3.772-27.017)	< 0.001

AKI=acute kidney injury; CI=confidence interval

**Table 3:** Comparison of clinic outcomes after propensity score matching (PSM) between transfusion (Transf.) group and control group.

Outcomes	After PSM			
	Transf. group (n=440)	Control group (n=440)	Odds ratio (95% CI)	*P*-value
Stroke	14 (3.2)	6 (1.4)	2.377 (0.905-6.244)	0.070
Myocardial infarction	4 (0.9)	2 (0.5)	2.009 (0.366-11.026)	0.413
Atrial fibrillation	16 (3.6)	9 (2)	1.807 (0.790-4.134)	0.156
Heart failure	10 (2.3)	3 (0.7)	3.388 (0.926-12.394)	0.050
AKI	6 (1.4)	3 (0.7)	2.014 (0.500-8.103)	0.315
Pulmonary infection	36 (8.2)	18 (4.1)	2.089 (1.167-3.739)	0.011
Sternal wound infection	7 (1.6)	2 (0.5)	3.540 (0.731-17.138)	0.180
Death within 30 days after surgery	12 (2.7)	1 (0.2)	12.308 (1.594-95.070)	0.002

AKI=acute kidney injury; CI=confidence interval

**Table 4:** Comparison of length of stay in intensive care unit (ICU), in-hospital stay, and death within 30 days after surgery before and after propensity score matching (PSM) between transfusion (Transf.) group and control group.

Outcomes	Before PSM			After PSM		
	Transf. group (n=631)	Control group (n=1547)	*P*-value	Transf. group (n=440)	Control group (n=440)	*P*-value
Mechanical ventilation (hours)	39.4±1.9	22.5±0.4	< 0.001	34.2±1.9	24.2±1.0	< 0.001
Length of stay in ICU (days)	2.36±0.12	1.41±0.02	< 0.001	2.02±0.09	1.51±0.05	< 0.001
Length of in-hospital stay after surgery (days)	8.04±0.16	6.57±0.05	< 0.001	7.63±0.14	6.98±0.12	< 0.001

ICU = Intensive care unit

Ischemic outcomes (including stroke and MI) increased in transfusion group. Adjusted OR estimates were 2.377 and 2.009 for postoperative stroke and MI, respectively. But there was no significant association with blood transfusion (*P*>0.05). Other complications (AF, HF, and AKI) in the transfusion patients also demonstrated an increasing tendency compared with non-transfusion patients. However, these connections had no practical significance in analysis after PSM (*P*>0.05).

Although the number of patients diagnosed with postoperative sternal wound infection in the transfusion group were more than in the non-transfusion group, it did not seem to be associated with blood transfusion (*P*>0.05). The increase of postoperative complications undoubtedly complicated the postoperative treatment process. Time of mean mechanical ventilation increased in the transfusion group compared to the non-transfusion group (34.2±1.9 hours and 24.2±1.0 hours, respectively). The mean of days of stay in ICU was 2.02±0.09 in the transfusion group compared with 1.51±0.05 in the non-transfusion group. The days of in-hospital stay after surgery were 7.63±0.14 in the transfusion group and 6.98±0.12 in the control group.

**Fig. 4:**
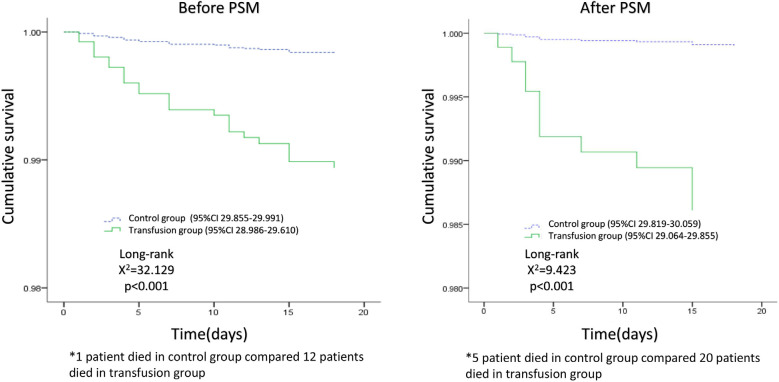
Comparison of cumulative survival within 30 days after surgery between transfusion group and control group. The cumulative survival within 30 days after surgery was lower in the transfusion group than in the non-transfusion group, both before and after propensity score matching (PSM) (P<0.001). CI=confidence interval.

The 30-day mortality of patients in the blood transfusion group was higher than in the control group (2.7% *vs*. 0.2%). The OR of 30-day mortality estimate associated with blood transfusion was 12.308. Kaplan-Meier survival curve of patients in both groups showed (Figure 4) that the 30-day cumulative survival rate in the transfusion group was lower than in the control group after PSM (log-rank test, *P*<0.05).

## DISCUSSION

The main finding of this study was that perioperative blood transfusion is significantly associated with postoperative pulmonary infection after adjusting for baseline covariates of preoperative and intraoperative factors (*P*<0.05). The risk of pulmonary infection in the transfusion group was approximately two-fold times higher than in the control group. Trend of patients diagnosed with pulmonary infection after surgery increased with the number of allogenenic RBC transfusion. There was a strong association of blood transfusion with pulmonary complication, especially when patients received > 4 units of allogeneic RBC transfusion.

As we know, patients undergoing CABG are commonly faced with risks for a variety of infections. Substantial hospital-level variation exists in postoperative hospital-acquired infections among patients undergoing CABG, driven predominantly by pneumonia^[5]^. Previous studies in literature have speculated that the cause of pulmonary infection after cardiac surgery is multifactorial. Allogeneic blood transfusion increased the risks of pulmonary complications, including pneumonia, transfusion-associated acute lung injury, and transfusion-associated dyspnea^[3,6]^. The reasons of these complications include bacterial infections, advanced age, renal failure (especially in dialysis patients), fluid overload, cardiac dysfunction, massive RBC infusion, and rapid infusion. Transfusion-related immunosuppression may also lead to susceptibility to microbial infection and down-regulation of cellular (T and natural killer cells) host defenses function^[7]^. Blood transfusion was also demonstrated to be related with predicting nosocomial pneumonia after CABG surgery^[8]^.

Other infections related to surgery also affect prognosis of patients. Secondary surgical-site infection after CABG continues to be an important source of morbidity^[9]^. This serious complication is associated with open saphenous vein graft harvesting, higher BMI, and blood transfusions. We observed the risk of sternal wound infection increased in the transfusion group compared with patients without transfusion after PSM. It will undoubtedly complicate the postoperative treatment process.

Increased postoperative pulmonary infection and other complications prolonged the duration of postoperative mechanical ventilation, length of stay in ICU, and in-hospital stay after surgery, and reduced short-term survival^[10]^. Patients with blood transfusion had significantly longer ICU stay and in-hospital stay in our study. We considered that prolonged days of stay in ICU and in-hospital stay were not necessarily directly related to blood transfusion. But the increase in postoperative complications associated with blood transfusion prolonged the duration of treatment in ICU and in hospital. Moreover, the mortality of the transfusion group within 30 days after surgery was significantly higher than in the non-transfusion group, which was consistent with previous study^[11]^. These factors may increase the consumption of patients during hospitalization, although the cost of hospitalization was not calculated in the study.

Minor transfusion of even one or two RBC units is associated with increased risks of several postoperative adverse events including AKI, sternal wound infection, postoperative use of antibiotics, prolonged use of pharmacological and mechanical inotropic support, length of ICU stay, and length of in-hospital stay^[12,13]^. AKI is a common and serious complication in heart surgery and increases morbidity and mortality. The incidence of cardiac surgery complicated by AKI varies between 7% and 40% in large cohorts undergoing a variety of cardiac surgeries. AKI after OPCABG was related to massive postoperative bleeding^[14]^. Blood transfusion was not necessarily associated with AKI, according to the results in our study. The incidence of AKI in this study was 1.4% in the transfusion group after propensity matching and demonstrated no significant difference in both groups. This result was different from a previous study and may benefited from OPCABG^[15]^. Actually, there were several potential pathophysiological causes of mechanisms of AKI after transfusion. The impairment of tissue oxygen delivery and predisposition to inflammatory response and oxidative stress promoted by transfusion might played an important role in organ injury^[16]^. Renal auto-regulation capability was impaired following ischemic injury and blood flow decreased with decreases in blood pressure. Previous study has also reported that it may be possible to help alleviate the incidence of AKI through an experienced multidisciplinary approach including intensivists, nephrologists, surgeons, and anesthesiologists^[17]^.

Postoperative cerebral ischemia was observed in this study. Patients diagnosed with a new stroke after OPCABG were approximately two-fold times higher in the transfusion group than in the control group. There are many causes of neurological complications after CABG, including continuous hypotension, prolonged extracorporeal circulation, hypoperfusion, and embolism caused by plaque shedding. Mikkola R. et al. considered that blood transfusion had a strong dose-dependent relationship with the risk of stroke after CABG^[18]^. It was suggested that the use of a heart fixture and platelet transfusion appeared to be associated with a greater risk of postoperative stroke than the use of RBC alone. The patients’ age, preoperative anemia, clopidogrel preoperative exposure, long-term use of warfarin, previous heart surgery, recent MI, emergency surgery, and critical state were associated with bleeding and transfusion in cardiac surgery; these risks that might be associated with postoperative neurological complications should still be considered^[1]^.

In addition, cardiac complications (including arrhythmia, MI, postoperative HF, etc.) are also important risks associated with postoperative adverse outcomes. The rates of postoperative AF, MI, and HF in patients who received blood transfusion increased when compared with non-transfusion patients in our study after adjusting preoperative and intraoperative confounders. Blood transfusion compared with non-transfusion was reported to be associated with higher all-cause mortality rates^[19]^. The relationship between transfusion and postoperative AF after CABG remains controversial. Previous meta-analysis reported a statistically significant increase in postoperative AF risk among adult patients with blood transfusion^[20]^. However, management measures to reduce perioperative blood transfusion such as restrictive RBC transfusion strategy was not superior to liberal strategy with respect to acute MI^[21]^. The correction of perioperative anemia was confirmed to be helpful in reducing the incidence of postoperative cardiac ischemic events^[22]^. Selection bias was considered to be an important reason for the inconsistency of many observational reports^[23]^. We considered that patients with ischemic heart disease may benefit from receiving blood transfusion, but this effect might be offset by some adverse reactions to allogeneic blood.

Because of the effect of allogeneic blood transfusion on the outcomes of surgical patients, many studies focused on how to reduce perioperative bleeding. Surgical factors were also considered to be related to perioperative blood transfusion, such as the number of anastomoses. The need for transfusion might be reduced by performing incomplete revascularization and making a strategy of contextualize hybrid coronary revascularization in treating with CABG^[24]^. In addition, perioperative use of non-steroidal anti-inflammatory drugs (NSAIDs) is often associated with postoperative bleeding. Discontinuation of NSAIDs before surgery may increase the risk of perioperative MI^[25]^. Therefore, preoperative use of NSAIDs in patients with CABG, should be considered comprehensively. Patients who discontinued aspirin at least seven days before surgery were adjusted in this study, so we could not conduct subgroup analysis of such patients. Further studies are needed to explain the relationship among NSAIDs, blood transfusions, and cardiac complications.

The results of this study confirmed the close relationship between clinical outcomes and blood transfusion in isolated OPCABG patients. We considered that the increased postoperative complications and blood transfusion may affected each other. The reasons affecting clinical outcomes in patients who underwent OPCABG are multifactorial. Comprehensive blood management strategy to reduce perioperative bleeding is helpful to reduce allogeneic transfusion^[1]^. In addition, the surgeon’s proficiency is also an important factor affecting perioperative bleeding. Appropriate reduction of allogeneic blood transfusion provides a feasible scheme for reducing postoperative complications and mortality.

### Limitations

Because errors due to differences caused by time and institutions were reduced, the results of this study may be different from other studies’ results, but still represent the factors most closely related to patients. There were several limitations in this study. Firstly, we noticed that the survival difference between the groups in this study seemed to be big. This kind of difference of early death was usually due to preoperative risk difference or postoperative complications. Although the statistical methods used in this study adjusted differences in baseline characteristics between the two groups, it was still possible that other risk factors associated with blood transfusion were not included in this study. Secondly, these results represented a single-center study. Despite the importance of the findings, the study has limitations because it is retrospective and has a short follow-up period. Perhaps a similar but prospective study could suggest that the patients included in the transfusion group were potentially more complex.

## CONCLUSION

Our study shows that perioperative blood transfusion increases the risks of postoperative pulmonary infection and short-term mortality in OPCABG patients.
